# Phenotypic plasticity to light and nutrient availability alters functional trait ranking across eight perennial grassland species

**DOI:** 10.1093/aobpla/plv029

**Published:** 2015-03-27

**Authors:** Alrun Siebenkäs, Jens Schumacher, Christiane Roscher

**Affiliations:** 1 Department of Community Ecology, Helmholtz Centre for Environmental Research -UFZ, Theodor-Lieser-Straße 4, 06120 Halle, Germany; 2 Institute of Stochastics, Friedrich Schiller University Jena, Ernst-Abbe-Platz 2, 07743 Jena, Germany

**Keywords:** Above- and belowground traits, forbs, functional groups, functional traits, grasses, growth stature, light, nutrients, trait variation

## Abstract

Functional traits are often used as species-specific mean trait values in comparative plant ecology or trait-based predictions of ecosystem processes, assuming that interspecific differences are greater than intraspecific trait variation and that trait-based ranking of species is consistent across environments. Although this assumption is increasingly challenged, there is a lack of knowledge regarding to what degree the extent of intraspecific trait variation in response to varying environmental conditions depends on the considered traits and the characteristics of the studied species to evaluate the consequences for trait-based species ranking. We studied functional traits of eight perennial grassland species classified into different functional groups (forbs vs. grasses) and varying in their inherent growth stature (tall vs. small) in a common garden experiment with different environments crossing three levels of nutrient availability and three levels of light availability over 4 months of treatment applications. Grasses and forbs differed in almost all above- and belowground traits, while trait differences related to growth stature were generally small. The traits showing the strongest responses to resource availability were similarly for grasses and forbs those associated with allocation and resource uptake. The strength of trait variation in response to varying resource availability differed among functional groups (grasses > forbs) and species of varying growth stature (small-statured > tall-statured species) in many aboveground traits, but only to a lower extent in belowground traits. These differential responses altered trait-based species ranking in many aboveground traits, such as specific leaf area, tissue nitrogen and carbon concentrations and above-belowground allocation (leaf area ratio and root : shoot ratio) at varying resource supply, while trait-based species ranking was more consistent in belowground traits. Our study shows that species grouping according to functional traits is valid, but trait-based species ranking depends on environmental conditions, thus limiting the applicability of species-specific mean trait values in ecological studies.

## Abstract (precis)

Functional traits are often used as species-specific means in trait-based predictions of ecosystem processes, assuming that interspecific differences are greater than intraspecific trait variation. We grew eight perennial grassland species representing two functional groups (grasses vs. forbs) and growth statures (small vs. tall) under different light and nutrient availability. The strength of trait variation in response to resource availability differed among functional groups and growth statures in many aboveground traits, while being more consistent in belowground traits. Our results, showing the dependency of trait-based species ranking on environmental conditions, limit the applicability of species-specific mean trait values in ecological studies.

## Introduction

There is a growing consensus that the use of functional traits has the potential to gain a better understanding of the functioning of organisms, how they relate to the environment and to address unresolved issues of community ecology and ecosystem research (Lavorel and Garnier 2002). It is mostly assumed that trait variation between species is much larger opposed to intraspecific trait variability (Diaz and Cabido 1997; McGill *et al.* 2006). This assumption is reflected in the a priori classification of plant species into functional groups, i.e. grouping of species according to similarities in their functional characteristics, as well as the application of more recently developed trait-based approaches (Lavorel *et al.* 1997; Dyer *et al.* 2001). However, both genetic differentiation and environmental variation are well-known factors, which may affect the phenotypic expression of functional traits (Coleman *et al.* 1994; Violle *et al.* 2012). In natural environments, plants are exposed to variation in multiple environmental factors and simultaneously compete for resources above- and belowground (Chapin *et al.* 1987). Trait variation at different levels of plant organization, ranging from physiological and biochemical to morphological characteristics, and allocation between plant organs enable plant species to adjust to a wide range of ecological conditions. Light availability and thus carbon acquisition via photosynthesis as well as soil nutrient availability are the most limiting factors for plant growth in temperate grasslands. Variation in traits associated with light acquisition and carbon assimilation, especially morphological and physiological leaf traits [e.g. specific leaf area (SLA) and leaf nitrogen concentrations (LNCs)], shoot traits associated with a better positioning of plant organs for light interception in dense canopies (e.g. height growth, allocation between leaves and supporting tissue) and biomass allocation between above- and belowground plant organs [root : shoot ratio (RSR) and leaf area ratio (LAR), i.e. leaf area per total dry mass] are typical responses to variation in light availability (Givnish 1988; Valladares and Niinemets 2008). In turn, variation in morphological root characteristics associated with nutrient uptake (e.g. specific root length (SRL), i.e. root length per unit root mass) and altered allocation between roots and shoots may result from changes in the availability of belowground resources (Ryser and Lambers 1995; Hill *et al.* 2006). However, different levels of nutrient availability may also induce an alteration in leaf morphological traits such as leaf dry matter content (LDMC) and SLA (Chapin *et al.* 1987; Hodgson *et al.* 2011). The close integration of plant carbon and nutrient metabolism requires a balance of various resources for growth. Thus, the acquisition of a single resource (e.g. carbon) is not independent of the availability of others (e.g. nutrients), and it is commonly assumed that plants allocate proportionally more resources to organs, which determine the capture of the most limiting resource to achieve a ‘functional equilibrium’ (Bloom *et al.* 1985; Poorter *et al.* 2012). An alternative explanation, however, is based on the observation that allocation into different plant organs is a function of plant size following non-linear allometric relationships (Coleman *et al.* 1994; Müller *et al.* 2000). Therefore, the degree of plasticity in changing allometric allocation is important to reduce resource imbalances (Shipley and Meziane 2002).

The most commonly accepted classification of non-legume herbaceous species into functional groups distinguishes between monocots (grasses) and dicots (forbs), mainly due to their taxonomy, phylogeny and differences in their growth forms (Dyer *et al.* 2001; Reich *et al.* 2003*a*). It has been shown that grasses have lower LNCs, greater leaf thickness and leaf tissue density, as well as smaller root diameters (RDs) and invest a smaller proportion of total biomass into leaves than forbs (Grime *et al.* 1997; Craine *et al.* 2001; Reich *et al.* 2003*b*; Poorter *et al*. 2012). Higher tissue density correlates with greater leaf longevity and plays a central role in plant strategies of nutrient acquisition and use (Grime *et al.* 1997; Aerts and Chapin 2000). Species with greater tissue density are thought to minimize nutrient loss and to maintain growth at low resource supply (= conservative species), while species with low tissue density are capable of fast resource acquisition and are more responsive in terms of growth to increased nutrient availability (= exploitative species) (Chapin 1980; Reich *et al.* 2003*b*). However, many ecological characteristics do not differ consistently between grasses and forbs, but display a large variation within these functional groups (Grime *et al.* 1997; Craine *et al.* 2001; Tjoelker *et al.* 2005). It has been hypothesized that exploitative species show greater trait plasticity than conservative species in response to nutrient availability (Crick and Grime 1987), but to our knowledge the few experimental studies testing this hypothesis on herbaceous species focussed exclusively on grass species and did not obtain consistent results (Van de Vijver *et al*. 1993; Grassein *et al*. 2010).

In temperate grasslands, usually a small number of species with an inherent taller growth achieves dominance and contributes the largest fraction of community biomass. A larger number of species with an inherent small growth stature is therefore more likely restricted to grow in low light conditions because taller growing species intercept a disproportionately larger fraction of light (Weiner 1990). Consequently, competition for light is asymmetric. Although soil resources are less likely pre-emptable, competition belowground has also been suggested to be size-asymmetric (Rajaniemi 2003). This size-asymmetry in competition raises the question how inherently tall- and small-statured species differ in their potential of responding plastically to varying light and nutrient availability.

Although it is well known that plants need to adjust to multiple local environmental factors, most experimental research focussing on functional trait variation to resource availability has manipulated single resources, such as nutrients (e.g. Al Haj Khaled *et al.* 2005; Mokany and Ash 2008; da Silveira Pontes *et al.* 2010; Kazakou *et al.* 2014) or light (Ryser and Eek 2000; Semchenko *et al.* 2012), while the interaction of light and nutrient availability on functional trait expression has rarely been studied or has been restricted to a smaller set of traits (Olff *et al.* 1990; Shipley and Almeida-Cortez 2003; Freschet *et al.* 2013). Previous studies manipulating single resources have shown that some aboveground traits are more plastic than others (Poorter *et al.* 2012; Kazakou *et al.* 2014) and that species ranking according to trait values is often consistent across environments (Mokany and Ash 2008; Kazakou *et al.* 2014). However, these results need to be extended to variation in multiple environmental factors covering species which may be assumed to play a different ecological role in natural communities and involving a larger set of traits.

In the present study, we grew eight perennial grassland species (four grasses, four forbs) of varying growth stature under controlled resource supply over 4 months at different combinations of light and nutrient availability to test the following hypotheses. (i) Grass species possess trait values associated with a more conservative use of resources and forb species show trait values indicative for a more exploitative use of resources, while differences between small- and tall-statured species in traits not directly related to height growth are small if grown as single plant individuals. (ii) The magnitude of trait variation in response to resource availability differs among traits and is greatest in traits related to allocation and the uptake of light and nutrients to maintain the ‘functional equilibrium’ at varying resource availability. (iii) The direction of trait variation in response to light and nutrient availability does not differ between grasses and forbs or small- and tall-statured species, but the extent of trait variation differs between forbs and grasses due to their different resource-use strategies. (iv) Trait-based species ranking varies across environments, but may be consistent for traits with similar plasticity across species or if differences in trait plasticity do not exceed interspecific trait differences.

## Methods

### Experimental design

Eight perennial species were chosen for the experiment. The four forb species and four grass species, including both small-statured and tall-statured species, are typical representatives of Central European semi-natural temperate grasslands (Molinio-Arrhenatheretea; Ellenberg 1988) (Table 1). Seeds were acquired from a commercial supplier (Rieger-Hoffman GmBH, Blaufelden-Raboldshausen, Germany). Seeds were pre-germinated in petri-dishes on moistened filter paper in an unheated glasshouse in April 2011. Seedlings in the stage of cotyledon emergence were separated and transferred into quickpots of 20 cm^3^ volume (Hermann Meyer KG, Rellingen, Germany). At the time of primary leaf emergence, single seedlings were transplanted into pots (volume 3 L, diameter 16.5 cm, height 18.5 cm) from 30 to 31 May 2011. Sieved topsoil from a close-by field (0–30 cm, czernozem; Altermann *et al.* 2005) at the experimental field station Bad Lauchstädt (Germany, 51°23′38″N, 11°52′45″E) was used as substrate [soil texture: loamy sand; pH 7.29; nitrogen 1.18 mg N g^−1^, carbonate 1.27 %, organic carbon 15.14 mg C g^−1^, C : N ratio 12.83, phosphorus (from double lactate extracts) 15.86 mg P kg^−1^, potassium (from calcium acetate lactate extracts) 51.7 mg K kg^−1^]. Plants were cultivated at ambient temperatures in a greenhouse with a roof, which automatically closes at rain.

**Table 1.** Studied species, plant height (Jäger 2011), grouping into functional groups (grasses vs. forbs) and growth stature (small vs. tall).

Species	Family	Height (cm)	Functional group	Stature
*Anthoxanthum odoratum* L.	Poaceae	20–50	Grass	Small
*Lolium perenne* L.	Poaceae	10–60	Grass	Small
*Arrhenatherum elatius* (L.) P. Beauv. ex J. Presl & C. Presl	Poaceae	60–120	Grass	Tall
*Dactylis glomerata* L.	Poaceae	50–120	Grass	Tall
*Plantago lanceolata* L.	Plantaginaceae	10–50	Forb	Small
*Prunella vulgaris* L.	Lamiaceae	5–30	Forb	Small
*Centaurea jacea ssp. jacea* L.	Asteraceae	15–80	Forb	Tall
*Knautia arvensis* (L.) Coulter	Dipsacaceae	30–80	Forb	Tall

Three weeks after transplanting, plants were randomly assigned to orthogonally crossed shade × fertilizer treatments of three levels each. The levels for shade treatments were full sunlight (= control), 40 % shade (= medium) and 70 % shade (= high), each replicated in six blocks arranged on tables (98 × 200 cm size). Shading was accomplished by fastening one (for 40 % shade) or two layers (for 70 % shade) of green shading cloth (polyethylene, aperture size 2 × 10 mm, Hermann Meyer KG, Rellingen, Germany) to aluminium frames at 96 cm height and closed on all sides. All plants received micronutrient solution [1 mL Hoagland A-Z solution, **see Supporting Information—Table S1**] and 1 mL of FeCl_3_ in 100 mL distilled water at the beginning of the experiment. NPK fertilization was applied equivalent to 62.5 mg nitrogen in total, spread over eight applications administered biweekly in 50 mL custom mixed fertilizer solution containing 6.9 g L^−1^ CaHPO_4_, 8.35 g L^−1^ K_2_SO_4_, 12.68 g L^−1^ MgSO_4_ · 7H_2_O and 3.57 g L^−1^ NH_4_NO_3_ for high-level fertilization, equivalent to 200 kg ha^−1^ year^−1^ nitrogen. Half the dosage was applied for medium-level fertilization, equivalent to 100 kg ha^−1^ year^−1^ nitrogen. These resemble the commonly applied fertilizer intensities in agriculturally managed semi-natural grasslands in Europe (Olff *et al.* 1990). Other plants did not receive additional nutrients throughout the whole duration of the experiment (= control).

Each species was cultivated with five replicates per treatment combination except for *Prunella vulgaris* L., for which one treatment combination (high shade × high fertilization) was lost due to seedling mortality. In total, 344 plants were grown. All plants were manually watered with tap water on a regular basis according to the estimated pot weight for 60 % water capacity and accounting for increasing plant size throughout the experiment. Plants assigned to different shading treatments were randomly assigned to blocks and re-arranged within blocks every 4 weeks.

### Data collection

Four months after initiating treatments with different light and nutrient availability, all plants were harvested from 10 to 20 October 2011. Shortly before the harvest, stomatal conductance (SC-1 Leaf Porometer, Decagon Devices Inc.) and leaf greenness (= unitless measure of foliar chlorophyll content; SPAD 502 Plus Chlorophyll Meter, Spectrum Technologies, Inc.) were measured (5–7 October 2011, between 10:00 and 15:00 h). Stomatal conductance was measured at one fully expanded leaf per individual using the auto mode of the porometer (taking the first 30 s of stomatal conductance data to predict the final stomatal conductance occurring under true steady state conditions). Leaf greenness was measured with five replicates on fully expanded leaves and averaged per individual. At the point of harvest, maximum (stretched) plant height was recorded. Plants were cut at ground level and stored overnight in a cooling chamber at 4 °C in wet paper towels to achieve water saturation. The following day, aboveground plant parts were separated into inflorescences, leaves and stems (including leaf sheaths in the case of grasses) and dead material (leaves with less than two-third of green tissue). Ten fully expanded leaves per plant were chosen, blotted dry using tissues to remove any surface water and immediately weighed. Then, leaf area of the bulk sample was measured with a LI-3100 Area Meter (LI-COR Inc., Lincoln, NE, USA). If grass individuals had more than 10 tillers, only 10 were randomly chosen for separation and measurements. At the time of harvest, single individuals (<10 %) of *A. elatius* and *K. arvensis* and ∼40 % of *P. lanceolata* plants had reached the reproductive stage. Belowground biomass was cleaned by rinsing off all soil over a 0.5 mm sieve. Root material was weighed and subsamples of 0.5–1 g fresh weight, representing the typical distribution of different root sizes of the plant individual, were stored at −20 °C. The root subsamples were thawed at a later point and scanned in deionized water on a flatbed scanner at 800 dpi and analysed with image analysis software (WinRHIZO; Regent Instruments, Quebec City, Canada). Root diameters calculated with this software are weighted by the overall length of analysed roots, thus attenuating the potential effect of thicker tap roots, if present. For each plant compartment, dry weight was determined after drying at 70 °C for 48 h. Total belowground biomass was derived by extrapolating dry mass of the scanned subsamples from the fresh mass to dry mass ratio of the remaining root system.

Three individuals per species and treatment combination were randomly selected for chemical analyses. Leaves (used for leaf area measurements), residual aboveground and root material of these individuals were separately chopped, finely ground with a ball mill and C and N concentrations were determined using an elemental analyser (Vario EL III Element Analyzer, Elementar, Hanau, Germany). All variables derived from these measurements are summarized in Table 2.

**Table 2.** Summary and description of variables investigated in this study.

Variable	Unit	Description	Variable group	Abbreviation
Specific leaf area	${{\text{m}}_{\mathrm{leaf}}}^{2}\;\text{k}{{\text{g}}_{\mathrm{leaf}}}^{-1}$	Leaf area per unit leaf dry mass	Leaf	SLA
Leaf dry matter content	$\text{m}{\text{g}}_{\mathrm{dw}\;\mathrm{leaf}}\;{{\text{g}}_{\mathrm{fw}\;\mathrm{leaf}}}^{-1}$	Leaf dry mass per water-saturated leaf fresh weight	Leaf	LDMC
Leaf nitrogen concentration	$\text{mg}\;\text{N}\;{{\text{g}}_{\mathrm{leaf}}}^{-1}$	Leaf nitrogen concentration	Leaf	LNC
Leaf carbon concentration	$\text{mg}\;\text{C}\;{{\text{g}}_{\mathrm{leaf}}}^{-1}$	Leaf carbon concentration	Leaf	LCC
Leaf greenness		Unitless measure of leaf chlorophyll concentration	Leaf	LeafG
Stomatal conductance	mmol m^−2^ s^−1^	Stomatal conductance per leaf area	Leaf	g_s_
Leaf mass fraction	${\text{g}}_{\mathrm{leaf}}{{\text{g}}_{\mathrm{shoot}}}^{-1}$	Leaf mass per aboveground shoot mass	Shoot	LMF
Shoot nitrogen concentration	$\text{mg}\;\text{N}\;{{\text{g}}_{\mathrm{shoot}}}^{-1}$	Shoot nitrogen concentration	Shoot	SNC
Shoot carbon concentration	$\text{mg}\;\text{C}\;{{\text{g}}_{\mathrm{shoot}}}^{-1}$	Shoot carbon concentration	Shoot	SCC
Plant height	cm	Stretched plant height	Shoot	Height
Leaf area ratio	mm_leaf_^2^ mg_plant_^−1^	Leaf area per total plant biomass	Allocation	LAR
Root:shoot ratio	g_root_ g^−1^_shoot_	Root biomass per aboveground biomass	Allocation	RSR
Root nitrogen concentration	$\text{mg}\;\text{N}\;{{\text{g}}_{\mathrm{root}}}^{-1}$	Root nitrogen concentration	Root	RNC
Root carbon concentration	$\text{mg}\;\text{C}\;{{\text{g}}_{\mathrm{root}}}^{-1}$	Root carbon concentration	Root	RCC
Specific root length	${\text{m}}_{\mathrm{root}}\;{{\text{g}}_{\mathrm{root}}}^{-1}$	Root length per root mass	Root	SRL
Root length density	cm_root_ cm_soil_^−3^	Root length per soil volume	Root	RLD
Root diameter	mm	Average root diameter	Root	RD
Total biomass	g	Total plant biomass	Performance	BM

### Data analysis

Data analysis was conducted with the statistical software R 3.0.2 (R Core Team 2013) including the package *lme4* (Bates *et al.* 2013). The software Canoco 4.5 (Biometris International, Wageningen) was used for multivariate analysis.

Linear mixed-effects models were used to determine to what extent differences among species assigned to different functional groups (forbs vs. grasses) and varying in growth stature (small vs. tall) as well as variation in resource availability (shade as linear term with 1 = full light, control, 2 = 40 % shade, 3 = 70 % shade; fertilizer as linear term with 1 = no fertilization, control, 2 = medium fertilization, 3 = high fertilization) and their interactions explained variation in the measured variables. Starting from a constant null model with block and species identity as random terms, the fixed effects were added stepwise in the following sequence: fertilization, shade, fertilization × shade interaction, functional group identity (grass vs. forb) and growth stature (tall vs. small) and their respective interaction with fertilization and shade. In order to evaluate the statistical significance of model improvement by sequential addition of fixed effects, the maximum likelihood method and likelihood ratio tests were applied. Data were transformed to logarithms to approach a better normal distribution except for stomatal conductance, leaf greenness, leaf N and C concentrations (no transformation), RSR (cubic root transformation) and height (square root transformation).

To decompose the variability attributable to model terms, mixed-effect models were fitted with the restricted maximum likelihood method. Variance components associated with random effects (block, species and residual) were estimated from the full model. To assess the fraction of variability associated with the fixed effects, a series of hierarchical models was fitted. For each individual effect, the share of explained variability was estimated as the difference between the total variability attributed to random effects in models not including and models including the respective fixed effect.

Resource availability is a critical determinant for inflorescence development and plant individuals must reach a critical size to initiate flowering in many herbaceous species (Sugiyama and Bazzaz 1998). To control for possible effects of plant developmental stage on trait differences, we introduced developmental stage (vegetative vs. reproductive) as a covariate before the experimental factors in additional models. The inclusion of developmental stage had only minor effects on the outcome of statistical analyses.

Additionally, the above-mentioned model was modified following a suggestion by Shipley and Meziane (2002) to test for non-linear allometric allocation in LAR and RSR as a function of plant size. The natural logarithm of total leaf area (or shoot biomass respectively) was modelled by additionally fitting the natural logarithm of total biomass (or root biomass, respectively) and its interaction with the previously described terms. The random term including species identity was modified by accounting for species-specific differences in the allometric relationship to plant size, i.e. the natural logarithm of total biomass and root biomass, respectively. Significant interactions of total biomass (or root biomass) with the experimental factors indicate deviations of the allometric slope dependent on resource availability, functional group identity or growth stature, while significance of the main experimental factors shows differences in the allometric intercept.

Furthermore, standardized principal component analysis (PCA) was applied to trait data of all species in combination to elucidate the major sources of variation in multiple traits. Data were corrected for block effects and transformed if necessary to achieve normal distribution before multivariate analyses. The resulting scores describing the distribution of plant individuals along the leading principal components were subjected to variance decomposition as described above for single traits.

The magnitude of intraspecific trait variation was estimated by calculating coefficients of variation (CVs, standard deviation over mean) across shade × nutrient treatments for each species based on mean values per treatment. In order to test for differences in the magnitude of trait variation between functional groups (grasses vs. forbs) and dependent on growth stature (tall vs. small), a two-factorial ANOVA was utilized. The consistency of species ranking in trait values across fertilizer × shade treatments was tested with Spearman’s rank correlation. A high correlation coefficient (*ρ* > 0.75, *n* = 36) resembles a consistent ranking of species independent of resource availability.

## Results

### Leaf traits

Fertilization had positive effects on LNCs and leaf greenness and negative effects on stomatal conductance, while fertilization did not impact SLA, LDMC and leaf carbon concentrations (LCCs) **[see Supporting Information****—Fig. S1A–F****]** (Table 3). Reduced light availability due to shading affected all leaf traits with exception of stomatal conductance (Table 3). Specific leaf area and LNC increased, while LDMC, LCC and leaf greenness decreased under shading **[see Supporting Information—Fig. S2A–F]** (Table 3). Nutrient and light availability did not interact in their effects on leaf traits.

**Table 3.** Summary of mixed-effects model analyses for functional traits combining all species. Models were fitted by stepwise inclusion of fixed effects. Likelihood ratio tests (*χ*^2^) were used to assess model improvement and the statistical significance of the explanatory terms (*P* values). For abbreviations and description of variables see Table 2.

	SLA		LDMC		LNC		LCC		LeafG		g_s_	
	*χ*^2^	*P*	*χ*^2^	*P*	*χ*^2^	*P*	*χ*^2^	*P*	*χ*^2^	*P*	*χ*^2^	*P*
Fertilizer	0.27	0.606	1.24	0.265	63.15	<0.001	1.25	0.263	87.91	<0.001	4.72	0.030
Shade	52.75	<0.001	32.84	<0.001	15.45	<0.001	6.43	0.011	31.63	<0.001	1.93	0.165
Fertilizer × shade	1.57	0.210	0.02	0.877	0.52	0.470	0.48	0.488	3.11	0.078	0.18	0.673
Functional group (FG)	1.14	0.285	14.34	<0.001	8.61	0.003	6.50	0.011	10.74	0.001	22.56	<0.001
FG × fertilizer	14.77	<0.001	9.27	0.002	0.93	0.334	1.60	0.206	21.49	<0.001	3.01	0.083
FG × shade	4.18	0.041	27.30	<0.001	1.54	0.215	0.78	0.378	70.01	<0.001	2.70	0.100
Growth stature (GS)	3.15	0.076	0.64	0.425	3.93	0.047	3.97	0.046	3.43	0.064	3.13	0.077
GS × fertilizer	1.15	0.284	0.89	0.346	0.37	0.543	1.04	0.309	0.89	0.344	0.18	0.671
GS × shade	0.67	0.413	6.82	0.009	2.96	0.085	10.24	0.001	0.36	0.550	4.08	0.043
FG × GS	0.32	0.575	2.22	0.136	1.67	0.196	3.94	0.047	0.15	0.701	5.77	0.016
	LMF		SNC		SCC		Height		LAR		RSR	
	*χ*^2^	*P*	*χ*^2^	*P*	*χ*^2^	*P*	*χ*^2^	*P*	*χ*^2^	*P*	*χ*^2^	*P*
Fertilizer	0.24	0.627	74.40	<0.001	0.71	0.401	18.58	<0.001	21.02	<0.001	121.78	<0.001
Shade	26.90	<0.001	27.60	<0.001	5.62	0.018	26.98	<0.001	56.29	<0.001	37.25	<0.001
Fertilizer × shade	3.85	0.050	0.67	0.411	5.66	0.017	0.00	0.980	0.85	0.357	3.53	0.060
Functional group (FG)	14.48	<0.001	17.69	<0.001	1.47	0.225	12.24	<0.001	1.48	0.224	0.77	0.380
FG × fertilizer	1.17	0.280	0.39	0.534	1.37	0.241	1.33	0.249	20.31	<0.001	13.30	<0.001
FG × shade	8.70	0.003	13.69	<0.001	5.27	0.022	6.14	0.013	13.66	<0.001	5.19	0.023
Growth stature (GS)	0.29	0.592	3.15	0.076	1.62	0.203	1.72	0.190	<0.01	0.951	8.93	0.003
GS × fertilizer	4.77	0.029	0.38	0.539	<0.01	0.972	2.45	0.118	0.04	0.842	8.99	0.003
GS × shade	15.03	<0.001	20.17	<0.001	<0.01	0.974	12.32	<0.001	5.84	0.016	0.23	0.631
FG × GS	1.02	0.313	5.34	0.021	0.35	0.552	2.05	0.152	0.35	0.554	2.96	0.085
	RNC		RCC		SRL		RLD		RD		Biomass	
	*χ*^2^	*P*	*χ*^2^	*P*	*χ*^2^	*P*	*χ*^2^	*P*	*χ*^2^	*P*	*χ*^2^	*P*
Fertilizer	68.00	<0.001	16.79	<0.001	33.72	<0.001	0.23	0.630	11.43	<0.001	47.68	<0.001
Shade	23.74	<0.001	5.06	0.024	29.12	<0.001	31.04	<0.001	2.64	0.104	30.61	<0.001
Fertilizer × shade	2.55	0.110	13.50	<0.001	0.70	0.404	1.93	0.165	0.89	0.347	0.38	0.539
Functional group (FG)	20.18	<0.001	0.61	0.435	4.61	0.032	14.67	<0.001	7.28	0.007	12.20	<0.001
FG × fertilizer	7.28	0.007	0.10	0.750	0.60	0.438	0.34	0.558	3.33	0.068	5.93	0.015
FG × shade	14.65	<0.001	<0.01	0.996	1.12	0.290	0.14	0.706	1.30	0.255	2.08	0.149
Growth stature (GS)	0.23	0.630	0.26	0.613	14.33	<0.001	1.20	0.274	2.98	0.084	0.17	0.684
GS × fertilizer	5.45	0.020	0.05	0.830	1.27	0.261	4.71	0.030	1.01	0.314	0.99	0.319
GS × shade	7.56	0.006	0.00	0.989	0.08	0.772	0.09	0.760	0.71	0.399	0.06	0.812
FG × GS	1.22	0.269	0.56	0.455	0.02	0.883	0.33	0.565	0.30	0.585	0.06	0.813

Grass and forb species differed in all leaf traits with the exception of SLA (Table 3). Forbs had greater LNC, leaf greenness and stomatal conductance and had lower LDMC and LCC than grasses. Variation in SLA, LDMC and leaf greenness in response to nutrient and light availability also differed between grasses and forbs. While in forb species SLA tended to decline and LDMC tended to increase with increasing fertilization, the opposite was observed in grass species. The positive effects of fertilization on leaf greenness were stronger in grasses than in forbs, whereas the decrease of leaf greenness in response to shading was more pronounced in forbs than in grasses. In contrast, the responsiveness of SLA (increase) and LDMC (decrease) to shading was more pronounced in grasses than in forbs. Leaf traits barely differed among species with tall and small growth stature, except for LNC and LCC, which were larger in tall-statured species. However, in small-statured species, shade had a stronger negative effect on LDMC and LCC. The opposite was the case for stomatal conductance (*g*_s_); *g*_s_ of tall-statured species showed a greater variation in response to shade than *g*_s_ of small-statured species.

### Shoot traits

Fertilization increased plant height and shoot nitrogen concentrations (SNCs), but did not affect leaf mass fraction (LMF) and shoot carbon concentrations (SCCs) **[see Supporting Information—Fig. S1G–J]** (Table 3). Shading decreased SCC and increased LMF, SNC and height **[see Supporting Information—Fig. S2G–J]**. Nutrient and light availability showed interactive effects on SCC. With both increasing nutrients and shade SCC declined. Forb and grass species differed in all aboveground shoot traits with exception of SCC. Grass species allocated less shoot mass to leaves (lower LMF), grew taller and had lower SNC than forb species. The effects of shading on shoot traits also differed between grasses and forbs. Grasses showed a stronger increase in LMF, SNC and height than forbs in response to shading, but shading led to a more pronounced reduction of SCC in forbs than in grasses. Aboveground shoot traits did not differ among species with tall and small growth stature, but the increase in plant height, SNC and LMF in response to shading was more pronounced in small-statured than in tall-statured species.

### Above- and belowground allocations

Fertilization as well as shading increased allocation in favour of aboveground plant organs [higher LAR, smaller RSR; **Supporting Information—Figs S1K and L and S2K and L**, Table 3]. Root : shoot ratio decreased with increasing shade because shading reduced root mass stronger than shoot mass. Nutrient and light availability did not interact in their effects on LAR and RSR. Above- and belowground allocations did not differ between functional groups, but the increase in LAR and the decrease in RSR in response to fertilization and shading were more pronounced in grasses than in forbs. Tall-statured species had a greater RSR than small-statured species, and fertilization led to a stronger decline in RSR in small-statured than in tall-statured species. In contrast, LAR did not depend on the growth stature. The increase in LAR in response to shading was more pronounced in small-statured than that in tall-statured species.

The inclusion of root mass in analyses of shoot mass to test for size-dependent variation in root : shoot allocation **[see Supporting Information—****Table S2****]** showed that the allometric slope varied between grasses and forbs [significant interaction FG × log(root mass)]: forbs had a steeper allometric slope (*α*) than grasses (*α*_forbs_ = 0.74 vs. *α*_grasses_ = 0.51). Different resource availability and growth stature, however, did not affect allometric slopes in root : shoot allocation. Testing the size dependency of variation in LAR **[see Supporting Information—Table S2]** rendered that the allometric slope varied dependent on nutrient availability, i.e. fertilization led to a steeper slope (*α*_control_ = 0.40 vs. *α*_fertilized_ = 0.78), while shading or differences between functional groups or growth statures did not affect allometric slopes.

### Belowground traits

Fertilization increased root nitrogen concentrations (RNCs) and RD and decreased SRL and root carbon concentrations (RCCs) **[see Supporting Information—Fig. S1M–Q]** (Table 3). Shading led to larger SRL and RNC, but resulted in lower root length density (RLD) and RCC **[see Supporting Information—Fig. S2M–Q]** (Table 3). Shading and fertilization interacted in their effects on RCC; in shade and at high nutrient availability RCC declined to the lowest levels.

Grass and forb species differed in all belowground traits except for RCC: forbs had lower SRL and RLD than grasses, but RNC and RD were greater in forbs than in grasses. The increase of RNC in response to shade was larger in grasses but the positive effect of fertilization on RNC was greater in forbs. Species of different growth stature differed in their SRL; small-statured species exhibited a higher SRL. Fertilization led to a larger increase in RLD in small-statured compared with tall-statured species. The effects of shading and fertilization on RNC were stronger in small-statured than in tall-statured species.

### Plant biomass

Performance expressed as total plant biomass increased with fertilization **[see Supporting Information—Fig. S1R]** and decreased with shading **[see Supporting Information—Fig. S2R]**, but both factors did not interact in their effects on total plant biomass (Table 3). Grass species produced more biomass than forbs, and positive effects of fertilization were more pronounced in grasses than in forbs. Total plant biomass did not differ among species with tall and small growth stature.

### Attribution of sources of variation in multiple traits

**Figure 1.**
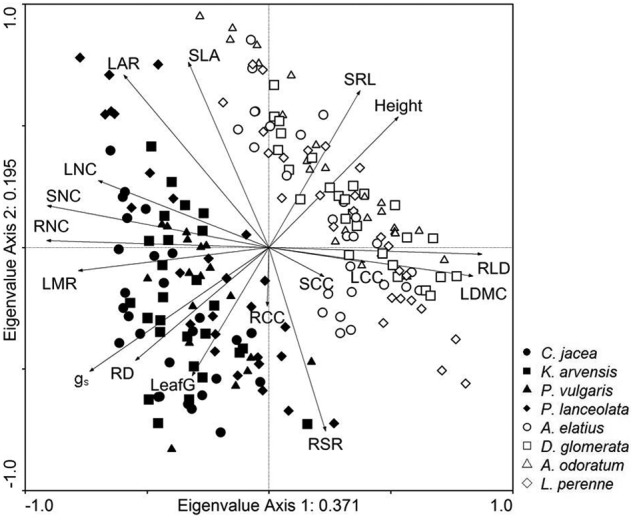
Standardized PCA of trait values across all studied species at different levels of resource availability. Abbreviations: SLA, specific leaf area; LDMC, leaf dry matter content; LNC, leaf nitrogen concentration; LCC, leaf carbon concentration; LeafG, leaf greenness; *g*_s_, stomatal conductance; LMF, leaf mass fraction; SNC, shoot nitrogen concentration; SCC, shoot carbon concentration; LAR, leaf area ratio; RSR, root : shoot ratio; RNC, root nitrogen concentration; RCC, root carbon concentration; RLD, root length density; SRL, specific root length; RD, root diameter.

The two leading axes of a standardized PCA of trait values across all species (Fig. 1) accounted for almost 60 % of variation. The first axis explaining 37 % of variation had high positive loadings for LDMC and RLD, opposed to high negative loadings for tissue N concentrations (SNC, RNC and LNC), LMF and stomatal conductance. The second axis explaining 19 % of variation had high positive loadings for SLA, LAR and SLR and high negative loadings for RSR. The major sources of variance explaining variation in multiple traits along the two leading axes were differences between functional groups (first axis 69 %, second axis 15 %) and due to shading (first axis 15 %, second axis 53 %) **[see Supporting Information—Fig. S3]**. The third axis mainly represented trait variation due to species identity (40 %) and fertilization (10 %).

### Magnitude of intraspecific trait variation

**Figure 2.**
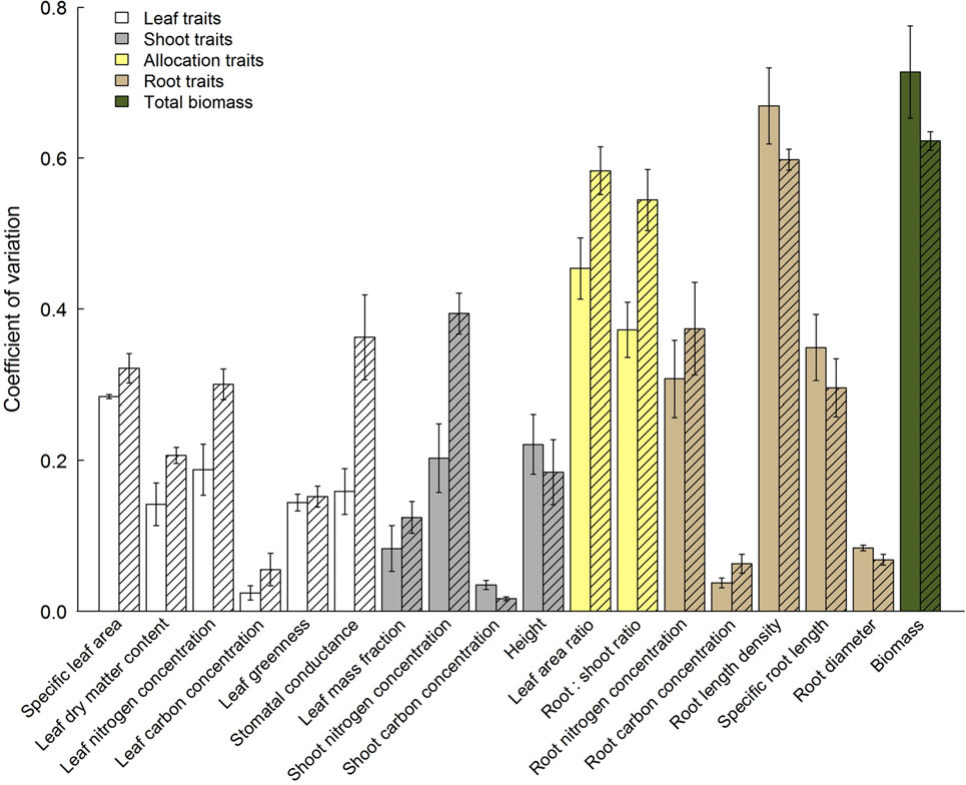
Intraspecific trait variation in response to resource availability estimated as coefficient of variation (CV, standard deviation over means) across treatments. Shown are means (±1 SE) across species. Hatched bars represent grass species.

Intraspecific trait variation quantified as CV across treatments differed greatly among traits, while differences among grasses vs. forbs and small vs. tall-statured species in intraspecific trait variation were often non-significant (Table 4). Overall, grasses had a greater intraspecific variation in stomatal conductance, LNC, LDMC, SNC and RSRs than forbs (Fig. 2). Small-statured species varied more in their LDMC and less in their leaf greenness than tall-statured species.

**Table 4.** Summary of two-factorial analysis of variance (ANOVA) of trait variation in response to resource availability estimated as coefficient of variation (CV, standard deviation over means) across treatments. Given are *F*-values and statistical significance of the explanatory terms (*P* values). For abbreviations and description of variables see Table 2.

	SLA		LDMC		LNC		LCC		LeafG		g_s_	
	*F*	*P*	*F*	*P*	*F*	*P*	*F*	*P*	*F*	*P*	*F*	*P*
Functional group (FG)	2.72	0.174	11.46	0.028	8.24	0.045	1.72	0.260	0.57	0.493	7.81	0.049
Growth stature (GS)	0.12	0.743	9.44	0.037	2.12	0.219	1.67	0.266	13.47	0.021	0.48	0.526
FG × GS	0.33	0.598	1.48	0.291	0.03	0.876	0.28	0.625	0.64	0.467	0.12	0.744
	LMF		SNC		SCC		LAR		RSR		Height	
	*F*	*P*	*F*	*P*	*F*	*P*	*F*	*P*	*F*	*P*	*F*	*P*
Functional group (FG)	1.45	0.295	24.43	0.008	6.56	0.063	5.42	0.080	14.62	0.019	0.62	0.475
Growth stature (GS)	2.24	0.209	7.10	0.056	1.14	0.346	1.01	0.371	2.71	0.175	5.43	0.080
FG × GS	0.78	0.427	0.11	0.753	<0.01	>0.999	0.10	0.769	2.15	0.216	0.06	0.814
	RNC		RCC		SRL		RLD		RD		Biomass	
	*F*	*P*	*F*	*P*	*F*	*P*	*F*	*P*	*F*	*P*	*F*	*P*
Functional group (FG)	1.02	0.371	2.20	0.212	0.79	0.426	1.26	0.324	3.40	0.139	2.04	0.227
Growth stature (GS)	4.78	0.094	0.04	0.858	0.02	0.897	0.04	0.848	1.02	0.369	1.01	0.372
FG × GS	0.02	0.889	0.06	0.815	1.59	0.276	<0.01	0.971	0.60	0.483	0.71	0.448

In general, carbon concentrations (SCC, LCC and RCC) showed the smallest intraspecific variation in response to different levels of resource availability (Fig. 2). Intraspecific variation in nitrogen concentrations (SNC, LNC and RNC) was greater, but comparable in different plant organs. Leaf dry matter content and leaf greenness were less variable than SLA, LNC and stomatal conductance among leaf traits, while LMF was less variable than height and SNC among shoot traits. Intraspecific variation in RNC and SRL were similar, but smaller than intraspecific variation in RLD. Characteristics related to above-belowground allocation (LAR and RSR), RLD as well as performance quantified as total biomass had the greatest intraspecific variation in response to varying resource availability.

### Attribution of sources of variation in single traits

**Figure 3.**
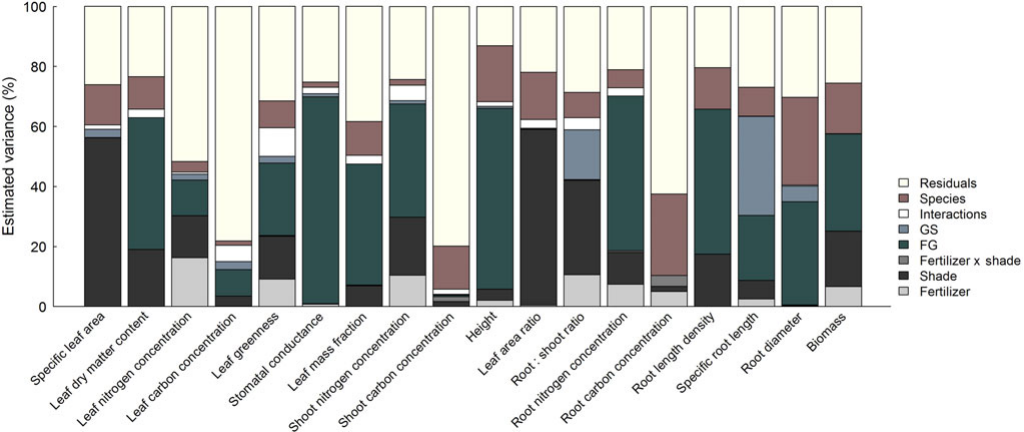
Estimated variance decomposition based on mixed-effects model analyses shown in Table 3. Note the variance components for block effects and residuals were combined in the graph, as well as the respective interactive effects of FG, GS × resource availability (as ‘interactions’).

Traits with a great plasticity in the response to shading were SLA, LAR (>50 % of variance), while the environment-induced variation in traits related to nitrogen-acquisition (LNC, leaf greenness, SNC and RNC), RSR and plant biomass were attributable to additive effects of shading and fertilization (>20 % of variance in total) (Fig. 3). A large proportion of variance in LDMC, leaf greenness, *g*_s_, plant biomass as well as all whole-shoot and root traits with exception of C concentrations was due to differences between functional groups. Differences due to growth statures mostly explained a minor proportion of variance with exception of RSR and SRL. Species identities often explained >10 % of residual variance with exception of tissue nitrogen concentrations, LCC and *g*_s_.

### Consistency of species ranking in trait values

**Figure 4.**
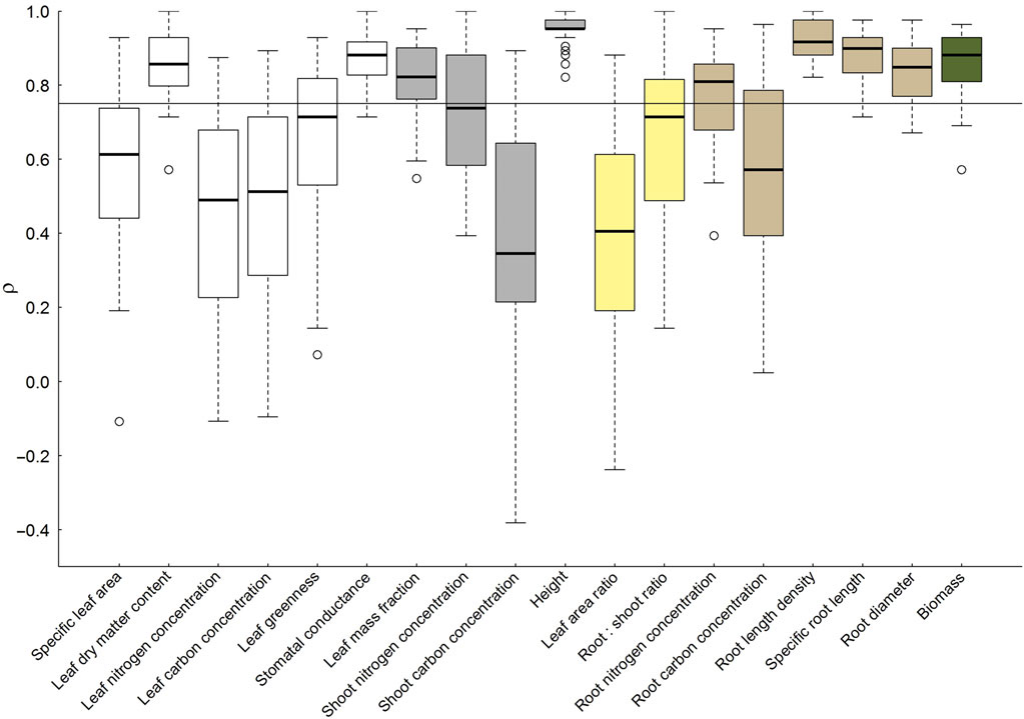
Species ranking in trait values across shade × fertilizer treatments, tested with Spearman’s rank correlation, where a high correlation coefficient (*ρ* > 0.75) indicates a consistent ranking of species independent of resource availability. For colours of trait groups see Figure 2.

Species ranking across all shade × fertilizer combinations remained conserved for LDMC, *g*_s_, plant height, root-morphological traits (RLD, SRL and RD) and plant biomass (Fig. 4). These traits had in common that >50 % of trait variation was attributable to the summed effects of functional group, growth stature and species identity (Fig. 3). The inconsistent ranking of species in other traits was mostly manifested through all treatment combinations, with exception of leaf greenness, where deviating species ranking was mainly caused by deep shade (not shown).

## Discussion

### Interspecific trait differences (Hypothesis 1)

The classification of grasses and forbs into distinct functional groups is widely applied in ecological studies. Grasses and forbs are known to differ in their shoot and root architecture and anatomy; however, it was repeatedly shown that this grouping may not be based on single characteristics, but that trait combinations including both above- and belowground traits distinguish between grasses and forbs most efficiently (Craine *et al*. 2001; Reich *et al*. 2003*a*; Tjoelker *et al*. 2005). Plant resource-use strategies may be described along a fundamental trade-off between resource acquisition and resource conservation and related to a ‘fast-slow’ plant economic spectrum (Reich 2014). High root N concentrations and SRLs are mechanistically related to root respiration, reflecting patterns of high metabolic activity associated with nutrient uptake and assimilation, and fast growth (Reich *et al.* 2003*a*; Tjoelker *et al*. 2005; Roumet *et al.* 2006). High leaf N concentrations correlate positively with leaf respiration and net photosynthesis rates and negatively with tissue longevity (e.g. Lambers and Poorter 1992; Reich *et al.* 2003*a*), while low tissue N concentrations indicate high nutrient retention (Aerts and Chapin 2000). In our study, grass species were characterized by greater LDMC, lower tissue N concentrations and had thinner roots, which is in line with our Hypothesis 1 that grasses possess trait combinations indicating a more conservative use of resources, while forbs were characterized by trait combinations associated with a more exploitative use of resources (greater tissue N concentrations, higher stomatal conductance and leaf greenness). In contrast, grass species had greater SRLs than forbs, which is supposed to be associated with an exploitative use of resources and greater rates of nutrient uptake. Species with ‘fast’ traits are assumed to grow best in higher resource conditions, while ‘slow’ species are thought to be superior when resource are scarce and conservation of resources results in better growth (Reich 2014). Nevertheless, grown as single plants, grass species accumulated more biomass than forb species in all treatments, which might be explained by greater costs for ‘fast’ traits of forb species. Trait differences between inherently small- and tall-statured species were generally small, which is also consistent with Hypothesis 1. Small-statured species had higher SRL, but invested less biomass into belowground organs (smaller RSR) than tall-statured species, resulting in comparable values for RLD. There was no evidence for differences in height growth and total biomass production after 4 months, showing that stature did not matter for growth when resource supply was externally controlled and not limited by competition. The greater RSR of tall-statured compared with small-statured species suggests that growth of tall species in their natural habitats is more likely limited by belowground resources (nutrients) and evolutionary processes selecting for greater investment into roots. It could be argued that differences in RSR were due to non-linear allometric allocation dependent on plant size (e.g. Shipley and Meziane 2002). Mixed-model analyses, however, showed that growth stature only affected the intercept of root : shoot allometry, while having no significant effects on the slope of this relationship **[see Supporting Information—Table S2]**.

### Effects of light and nutrient availability on trait expression (Hypothesis 2)

In accordance with results from earlier studies, shading resulted in greater investment into aboveground plant organs (higher LAR and lower RSR), higher tissue N concentrations, but lower tissue C concentrations (lower C/N ratios due to reduced growth) and the formation of leaves with larger SLA (Ryser and Lambers 1995; Ryser and Eek 2000; Evans and Poorter 2001). Less is known about the effect of shade on belowground morphology. Ryser and Eek (2000) showed that SRL of *D. glomerata* increased, when light availability was reduced to 20–30 % of full daylight, which is consistent with our results. A better nutrient availability is known to increase allocation to aboveground plant organs (higher LAR and lower RSR) (Elberse and Berendse 1993; Ryser and Lambers 1995). Root-morphological changes, i.e. a decreased RD and increased SRL, have been described in response to phosphorus deficiency, but less concordant effects have been observed for nitrogen deficiency (Hill *et al.* 2006). In our study, using NPK fertilization, we found decreasing SRL and increasing RD with fertilization in congruence with other studies (Fransen *et al.* 1999; Grossman and Rice 2012). Despite shading- or fertilization-induced plastic changes in all studied traits (Table 3), the magnitude of trait responsiveness varied greatly among traits. Allocation between above- and belowground plant organs (LAR and RSR), traits related to resource uptake (SLA and SRL) and RLD, which is closely related with SRL, were the most plastic characteristics in all studied species (Fig. 2) as predicted by our Hypothesis 2. However, plasticity in tissue nitrogen concentrations also showed considerable variation in response to resource availability suggesting that C and nutrient metabolism were not balanced at different levels of resource supply.

### Extent and structure of trait variation as affected by functional groups and growth stature (Hypothesis 3)

Albeit similar responses across all studied species, significant interactions between functional group identity and resource availability (shade, fertilization) in several leaf traits (SLA, LDMC and leaf greenness), above- and belowground allocations (RSR and LAR), height and shoot C and N concentrations showed that aboveground traits of grasses and forbs differed in their responsiveness to environmental variation as suggested by our Hypothesis 3, while this was not the case for root-morphological traits (Table 3). Although studies comparing pairs of grass species from fertile and infertile habitats suggested that exploitative species show greater plasticity to nutrient availability than conservative species (Crick and Grime 1987; Grassein *et al*. 2010), we found that grass species having traits characteristic for a conservative use of resources were more plastic in several traits (LDMC, LNC, *g*_s_, SNC and RSR) than forb species (Fig. 2, Table 4). In accordance with our expectations, we also found that trait variation of small-statured species in response to light availability was greater than in tall-statured species in many aboveground traits, suggesting that inherently small species, which often suffer from light competition in their natural habitats, have been selected for greater responsiveness to shade (Table 3).

### Effects of light and nutrients on trait-based species ranking (Hypothesis 4)

A number of recent studies have pointed out that intraspecific trait variation might be equally important to consider in ecological studies than interspecific trait differences (Albert *et al.* 2011; Violle *et al.* 2012). The consistency of trait-based species ranking in different environments depends on the direction of trait variation in response to environmental variation and the relative magnitude of inter- vs. intraspecific variation (Garnier *et al.* 2001). Several studies, mostly conducted at varying nutrient supply, delivered heterogeneous results. For example, consistent species rankings have been found for leaf traits such as SLA and LDMC (Al Haj Khaled *et al.* 2005; Mokany and Ash 2008; Kazakou *et al.* 2014), while Rose *et al.* (2013) reported less consistent species rankings in these traits. However, it has also been emphasized that the stability of species rankings depends on the considered trait (Kazakou *et al.* 2014). Our study manipulating nutrient supply in combination with different levels of shading, provided clear evidence that trait-based species ranking is only consistent across environments, when trait variation in response to resource availability is small compared with differences between functional groups, growth statures or dependent on species identity (Figs 2 and 3) or if the magnitude and direction of trait variation does not differ between species, confirming Hypothesis 4. For example, species ranks in all root-morphological traits (RLD, SRL and RD) were largely consistent although these traits differed greatly in their intraspecific variation in response to resource availability (Fig. 3), but plasticity in these traits did not differ between functional groups or dependent on growth stature (Table 3). In contrast, species ranking in LDMC and plant height was also consistent, probably due to great differences in trait values between species buffering against the differential effects of resource availability dependent on functional group identity or growth statures.

## Conclusions

The usefulness of trait-based definitions of plant functional groups depends on their repeatability (Gitay and Noble 1997) implying that environment-induced variation in trait expression is similar across species. Our study showed that species assigned to the predefined functional groups of grasses and forbs differed in most studied traits. The differentiation between grasses and forbs based on multiple traits remained robust irrespective of light and nutrient availability (Fig. 1), and the identity of traits most responsive to variation in resource availability was similar among grasses and forbs (Fig. 2) justifying species classification into the commonly used functional groups (Dyer *et al.* 2001; Tjoelker *et al.* 2005). However, small interspecific differences in combination with varying plasticity in response to the environmental conditions altered trait-based ranking among species in several traits. The varying consistency in trait-based ranking may limit the usefulness of functional groups as well as the applicability of species-specific mean trait values in predicting species or community responses to environmental variation.

## Sources of Funding

This work was supported by the German Science Foundation (RO2397/4).

## Contributions by the Authors

All authors designed the experiment, A.S. and C.R. performed the experiment, A.S., J.S. and C.R. analysed the data, A.S. drafted the manuscript and C.R. and J.S. contributed to writing the manuscript.

## Conflict of Interest Statement

None declared.

## Supporting Information

The following additional information is available in the online version of this article –

**Table S1.** Composition of micronutrient solution.

**Table S2.** Summary of mixed-effects model analyses across all studied species testing for non-linear allometric allocation in leaf area ratio and root to shoot ratio.

**Figure S1.** Trait values of eight studied grassland species in response to three different levels of fertilization (control = no fertilizer addition, medium and high).

**Figure S2.** Trait values of eight studied grassland species in response to different levels of shade (control = full light, medium and high).

**Figure S3.** Estimated variance decomposition based on mixed-effects model analyses for leading principal components based on multiple traits.

Additional Information

## References

[PLV029C1] Aerts R Chapin FS 2000 The mineral nutrition of wild plants revisited: a re-evaluation of processes and patterns. Advances in Ecological Research 30:1–67. 10.1016/S0065-2504(08)60016-1

[PLV029C2] Albert CH Grassein F Schurr FM Vieilledent G Violle C 2011 When and how should intraspecific variability be considered in trait-based plant ecology? Perspectives in Plant Ecology, Evolution and Systematics 13:217–225. 10.1016/j.ppees.2011.04.003

[PLV029C3] Al Haj Khaled R Duru M Theau JP Plantureux S Cruz P 2005 Variation in leaf traits through seasons and N-availability levels and its consequences for ranking grassland species. Journal of Vegetation Science 16:391–398. 10.1111/j.1654-1103.2005.tb02378.x

[PLV029C4] Altermann M Rinklebe J Merbach I Körschens M Langer U Hofmann B 2005 Chernozem—soil of the year 2005. Journal of Plant Nutrition and Soil Science 168:725–740. 10.1002/jpln.200521814

[PLV029C5] Bates D Maechler M Bolker B 2013 lme4: linear mixed-effects models using S4 classes. http://www.r-project.org.

[PLV029C6] Bloom AJ Chapin FS Mooney HA 1985 Resource limitation in plants—an economic analogy. Annual Review of Ecology and Systematics 16:363–392.

[PLV029C7] Chapin FS 1980 The mineral nutrition of wild plants. Annual Review of Ecology and Systematics 11:233–280. 10.1146/annurev.es.11.110180.001313

[PLV029C8] Chapin FS Bloom AJ Field CB Waring RH 1987 Plant responses to multiple environmental factors. BioScience 37:49–57. 10.2307/1310177

[PLV029C9] Coleman JS McConnaughay KDM Ackerly DD 1994 Interpreting phenotypic variation in plants. Trends in Ecology and Evolution 9:187–191. 10.1016/0169-5347(94)90087-6 21236817

[PLV029C10] Craine JM Froehle J Tilman DG Wedin DA Chapin FS 2001 The relationships among root and leaf traits of 76 grassland species and relative abundance along fertility and disturbance gradients. Oikos 93:274–285. 10.1034/j.1600-0706.2001.930210.x

[PLV029C11] Crick JC Grime JP 1987 Morphological plasticity and mineral nutrient capture in two herbaceous species of contrasted ecology. New Phytologist 107:403–414. 10.1111/j.1469-8137.1987.tb00192.x 33873852

[PLV029C12] da Silveira Pontes L Louault F Carrère P Maire V Andueza D Soussana J-F 2010 The role of plant traits and their plasticity in the response of pasture grasses to nutrients and cutting frequency. Annals of Botany 105:957–965. 10.1093/aob/mcq066 20354073 PMC2876009

[PLV029C13] Diaz S Cabido M 1997 Plant functional types and ecosystem function in relation to global change. Journal of Vegetation Science 8:463–474. 10.1111/j.1654-1103.1997.tb00842.x

[PLV029C14] Dyer AR Goldberg DE Turkington R Sayre C 2001 Effects of growing conditions and source habitat on plant traits and functional group definition. Functional Ecology 15:85–95. 10.1046/j.1365-2435.2001.00487.x

[PLV029C15] Elberse WT Berendse F 1993 A comparative study of the growth and morphology of eight grass species from habitats with different nutrient availabilities. Functional Ecology 7:223–229. 10.2307/2389891

[PLV029C16] Ellenberg H 1988 Vegetation ecology of Central Europe. Cambridge, UK: Cambridge University Press.

[PLV029C17] Evans JR Poorter H 2001 Photosynthetic acclimation of plants to growth irradiance: the relative importance of specific leaf area and nitrogen partitioning in maximizing carbon gain. Plant, Cell and Environment 24:755–767. 10.1046/j.1365-3040.2001.00724.x

[PLV029C18] Fransen B Blijjenberg J de Kroon H 1999 Root morphological and physiological plasticity of perennial grass species and the exploitation of spatial and temporal heterogeneous nutrient patches. Plant and Soil 211:179–189. 10.1023/A:1004684701993

[PLV029C19] Freschet GT Bellingham PJ Lyver PO Bonner KI Wardle DA 2013 Plasticity in above- and belowground resource acquisition traits in response to single and multiple environmental factors in three tree species. Ecology and Evolution 3:1065–1078. 10.1002/ece3.520 23610644 PMC3631414

[PLV029C20] Garnier E Laurent G Bellmann A Debain S Berthelier P Ducout B Roumet C Navas M-L 2001 Consistency of species ranking based on functional leaf traits. New Phytologist 152:69–83. 10.1046/j.0028-646x.2001.00239.x 35974476

[PLV029C21] Gitay H Noble IR 1997 What are functional types and how should we seek for them? In: Smith TM Shugart HH Woodward FI, eds. Plant functional types—their relevance to ecosystem properties and global change. Cambridge: Cambridge University Press, 3–19.

[PLV029C22] Givnish TJ 1988 Adaptation to sun and shade: a whole-plant perspective. Australian Journal of Plant Physiology 15:63–92. 10.1071/PP9880063

[PLV029C23] Grassein F Till-Bottraud I Lavorel S 2010 Plant resource-use strategies: the importance of phenotypic plasticity in response to a productivity gradient for two subalpine species. Annals of Botany 106:637–645. 10.1093/aob/mcq154 20682576 PMC2944977

[PLV029C24] Grime JP Thompson K Hunt R Hodgson JG Cornelissen JHC Rorison IH Hendry GAF Ashenden TW Askew AP Band SR Booth RE Bossard CC Campbell BD Cooper JEL Davison AW Gupta PL Hall W Hand DW Hannah MA Hillier SH Hodkinson DJ Jalili A Liu Z Mackey JML Matthews N Mowforth MA Neal AM Reader RJ Reiling K Ross-Fraser W Spencer RE Sutton F Tasker DE Thorpe PC Whitehouse J 1997 Integrated screening validates primary axes of specialisation in plants. Oikos 79:259–281. 10.2307/3546011

[PLV029C25] Grossman JD Rice KJ 2012 Evolution of root plasticity responses to variation in soil nutrient distribution and concentration. Evolutionary Applications 5:850–857. 10.1111/j.1752-4571.2012.00263.x 23346229 PMC3552402

[PLV029C26] Hill JO Simpson RJ Moore AD Chapman DF 2006 Morphology and response of roots of pasture species to phosphorus and nitrogen nutrition. Plant and Soil 286:7–19. 10.1007/s11104-006-0014-3

[PLV029C27] Hodgson JG Montserrat-Martí G Charles M Jones G Wilson P Shipley B Sharafi M Cerabolini BEL Cornelissen JHC Band SR Bogard A Castro-Diez P Guerrero-Campo J Palmer C Perez-Rontome MC Carter G Hynd A Romo-Diez A de Torres Espuny L Royo Pla F 2011 Is leaf dry matter content a better predictor of soil fertility than specific leaf area? Annals of Botany 108:1337–1345. 10.1093/aob/mcr225 21948627 PMC3197453

[PLV029C28] Jäger EJ, ed. 2011 Rothmaler. Exkursionsflora von Deutschland. Gefäßpflanzen: Grundband. Heidelberg: Spektrum.

[PLV029C29] Kazakou E Violle C Roumet C Navas M-L Vile D Kattge J Garnier E 2014 Are trait-based species rankings consistent across data sets and spatial scales? Journal of Vegetation Science 25:235–247. 10.1111/jvs.12066

[PLV029C30] Lambers H Poorter H 1992 Inherent variation in growth rate between higher plants: a search for physiological causes and ecological consequences. Advances in Ecological Research 22:187–261.

[PLV029C31] Lavorel S Garnier E 2002 Predicting changes in community composition and ecosystem functioning from plant traits: revisiting the Holy Grail. Functional Ecology 16:545–556. 10.1046/j.1365-2435.2002.00664.x

[PLV029C32] Lavorel S McIntyre S Landsberg J Forbes TDA 1997 Plant functional classifications: from general groups to specific groups based on response to disturbance. Trends in Ecology and Evolution 12:474–478. 10.1016/S0169-5347(97)01219-6 21238163

[PLV029C33] McGill BJ Enquist BJ Weiher E Westoby M 2006 Rebuilding community ecology from functional traits. Trends in Ecology and Evolution 21:178–185. 10.1016/j.tree.2006.02.002 16701083

[PLV029C34] Mokany K Ash J 2008 Are traits measured on pot grown plants representative of those in natural communities? Journal of Vegetation Science 19:119–126. 10.3170/2007-8-18340

[PLV029C35] Müller I Schmid B Weiner J 2000 The effect of nutrient availability on biomass allocation patterns in 27 species of herbaceous plants. Perspectives in Plant Ecology, Evolution and Systematics 3:115–127. 10.1078/1433-8319-00007

[PLV029C36] Olff H Andel JV Bakker JP 1990 Biomass and shoot/root allocation of five species from a grassland succession series at different combinations of light and nutrient supply. Functional Ecology 4:193–200. 10.2307/2389338

[PLV029C37] Poorter H Niklas KJ Reich PB Oleksyn J Poot P Mommer L 2012 Biomass allocation to leaves, stems and roots: meta-analyses of interspecific variation and environmental control. New Phytologist 193:30–50. 10.1111/j.1469-8137.2011.03952.x 22085245

[PLV029C55] R Core Team. 2013 R: A language and environment for statistical computing. R Foundation for Statistical Computing. Vienna, Austria http://www.R-project.org/.

[PLV029C38] Rajaniemi TK 2003 Evidence for size asymmetry of belowground competition. Basic and Applied Ecology 4:239–247. 10.1078/1439-1791-00151

[PLV029C39] Reich PB 2014 The world-wide ‘fast-slow’ plant economics spectrum: a traits manifesto. Journal of Ecology 102:275–301. 10.1111/1365-2745.12211

[PLV029C40] Reich PB Buschena C Tjoelker MG Wrage K Knops J Tilman D Machado JL 2003a Variation in growth rate and ecophysiology among 34 grassland and savanna species under contrasting N supply: a test of functional group differences. New Phytologist 157:617–631. 10.1046/j.1469-8137.2003.00703.x 33873411

[PLV029C41] Reich PB Wright IJ Cavender-Bares J Craine JM Oleksyn J Westoby M Walters MB 2003b The evolution of plant functional variation: traits, spectra, and strategies. International Journal of Plant Sciences 164:S143–S164. 10.1086/374368

[PLV029C42] Rose L Rubarth MC Hertel D Leuschner C 2013 Management alters interspecific leaf trait relationships and trait-based species rankings in permanent meadows. Journal of Vegetation Science 24:239–250. 10.1111/j.1654-1103.2012.01455.x

[PLV029C43] Roumet C Urcelay C Díaz S 2006 Suites of root traits differ between annual and perennial species growing in the field. New Phytologist 170:357–368. 10.1111/j.1469-8137.2006.01667.x 16608460

[PLV029C44] Ryser P Eek L 2000 Consequences of phenotypic plasticity vs. interspecific differences in leaf and root traits for acquisition of aboveground and belowground resources. American Journal of Botany 87:402–411. 10719001

[PLV029C45] Ryser P Lambers H 1995 Root and leaf attributes accounting for the performance of fast- and slow-growing grasses at different nutrient supply. Plant and Soil 170:251–265. 10.1007/BF00010478

[PLV029C46] Semchenko M Lepik M Götzenberger L Zobel K 2012 Positive effect of shade on plant growth: amelioration of stress or active regulation of growth rate? Journal of Ecology 100:459–466. 10.1111/j.1365-2745.2011.01936.x

[PLV029C47] Shipley B Almeida-Cortez J 2003 Interspecific consistency and intraspecific variability of specific leaf area with respect to irradiance and nutrient availability. Ecoscience 10:74–79.

[PLV029C48] Shipley B Meziane D 2002 The balanced-growth hypothesis and the allometry of leaf and root biomass allocation. Functional Ecology 16:326–331. 10.1046/j.1365-2435.2002.00626.x

[PLV029C49] Sugiyama S Bazzaz FA 1998 Size dependence of reproductive allocation: the influence of resource availability, competition and genetic identity. Functional Ecology 12:280–288. 10.1046/j.1365-2435.1998.00187.x

[PLV029C50] Tjoelker MG Craine JM Wedin D Reich PB Tilman D 2005 Linking leaf and root trait syndromes among 39 grassland and savannah species. New Phytologist 167:493–508. 10.1111/j.1469-8137.2005.01428.x 15998401

[PLV029C51] Valladares F Niinemets Ü 2008 Shade tolerance, a key plant feature of complex nature and consequences. Annual Review of Ecology, Evolution, and Systematics 39:237–257. 10.1146/annurev.ecolsys.39.110707.173506

[PLV029C52] Van de Vijver CADM Boot RGA Poorter H Lambers H 1993 Phenotypic plasticity in response to nitrate supply of an inherently fast-growing species from a fertile habitat and an inherently slow-growing species from an infertile habitat. Oecologia 96:548–554. 10.1007/BF00320512 28312461

[PLV029C53] Violle C Enquist BJ McGill BJ Jiang L Albert CH Hulshof C Jung V Messier J 2012 The return of the variance: intraspecific variability in community ecology. Trends in Ecology and Evolution 27:244–252. 10.1016/j.tree.2011.11.014 22244797

[PLV029C54] Weiner J 1990 Asymmetric competition in plant populations. Trends in Ecology and Evolution 5:360–364. 10.1016/0169-5347(90)90095-U 21232393

