# Supplementary material for: Phenotypic plasticity to light and nutrient availability alters functional trait ranking across eight perennial grassland species
Source: AoB Plants. 2015 Mar 27;7:plv029. doi: 10.1093/aobpla/plv029 (PMC4417138; doi:10.1093/aobpla/plv029)
Supplement: Additional Information [file supp_7_plv029_index.html]

Phenotypic plasticity to light and nutrient availability alters functional trait ranking across eight perennial grassland species — Additional Information 

# Phenotypic plasticity to light and nutrient availability alters functional trait ranking across eight perennial grassland species

## Additional Information

Additional Information

**Files in this Data Supplement:**

- Additional Information - Doc file
