# Supplementary material for: Phenotypic plasticity to light and nutrient availability alters functional trait ranking across eight perennial grassland species
Source: AoB Plants. 2015 Mar 27;7:plv029. doi: 10.1093/aobpla/plv029 (PMC4417138; doi:10.1093/aobpla/plv029)
Supplement: Additional Information [file supp_plv029_plv029supp.doc]

# SUPPORTING INFORMATION

Table S1. Summary of chemical concentrations for Hoagland A-Z micronutrient solution applied before the beginning of the experiment

| Component solution a) | mg l^-1^ | Component solution b) | mg l^-1^ |
| --- | --- | --- | --- |
| Al_2_(SO_4_)_2_ | 56 | As_2_O_3_ | 5.6 |
| B(OH)_3_ | 611 | BaCl_2_ | 33 |
| Co(NO_3_)_2_ x 6 H_2_O | 56 | CdCl_2_ | 5.6 |
| CuSO4 x 5 H_2_O | 56 | Bi(NO_3_)_3_ | 6.8 |
| KBr | 28 | RbSO_4_ | 5.6 |
| KI | 28 | K_2_CrO_4_ | 28 |
| LiCl | 28 | KF | 5.6 |
| MnCl_2_ x 4 H_2_O | 389 | PbCl_2_ | 5.6 |
| NiSO_4_ x 7 H_2_O | 56 | HgCl_2_ | 5.6 |
| SnCl_2_ x 2 H_2_O | 28 | MoO_3_ | 28 |
| TiO_2_ | 56 | H_2_SeO_4_ | 5.6 |
| ZnSO_4_ x 7 H_2_O | 99 | SeSO_4_ | 28 |
|  |  | HWO_4_ | 5.6 |
|  |  | VCl_3_ | 7.2 |

Table S2. Summary of mixed-effects model analyses across all studied species testing for non-linear allometric allocation in leaf area ratio (relating leaf area to total biomass) and root:shoot ratio (relating shoot biomass to root biomass)

|  | Leaf area | |  |  | Shoot biomass | |
| --- | --- | --- | --- | --- | --- | --- |
|  | χ^2^ | P |  |  | χ^2^ | P |
| Fertilizer | 46.73 | <0.001 |  | Fertilizer | 181.18 | <0.001 |
| Shade | 54.76 | <0.001 |  | Shade | 13.73 | <0.001 |
| Functional group (FG) | 1.09 | 0.300 |  | Functional group (FG) | 3.79 | 0.051 |
| Growth stature (GS) | 3.16 | 0.075 |  | Growth stature (GS) | 9.34 | 0.002 |
| Total biomass (BM) | 35.84 | <0.001 |  | Root biomass (BM) | 28.08 | <0.001 |
| Fertilizer x Shade | 2.30 | 0.129 |  | Fertilizer x Shade | 5.66 | 0.017 |
| FG x Fertilizer | 35.12 | <0.001 |  | FG x Fertilizer | 27.11 | <0.001 |
| FG x Shade | 5.99 | 0.014 |  | FG x Shade | 0.15 | 0.700 |
| GS x Fertilizer | 0.44 | 0.508 |  | GS x Fertilizer | 8.63 | 0.003 |
| GS x Shade | 7.37 | 0.007 |  | GS x Shade | 0.07 | 0.791 |
| Fertilizer x Total BM | 10.45 | 0.001 |  | Fertilizer x Root BM | 3.51 | 0.060 |
| Shade x Total BM | 1.04 | 0.309 |  | Shade x Root BM | 2.60 | 0.107 |
| FG x Total BM | 0.97 | 0.325 |  | FG x Root BM | 5.96 | 0.014 |
| GS x Total BM | 0.45 | 0.502 |  | GS x Root BM | 0.03 | 0.870 |

Models were fitted by stepwise inclusion of fixed effects. Likelihood ratios tests (χ^2^) were used to assess model improvement and the statistical significance of the explanatory terms (P values).


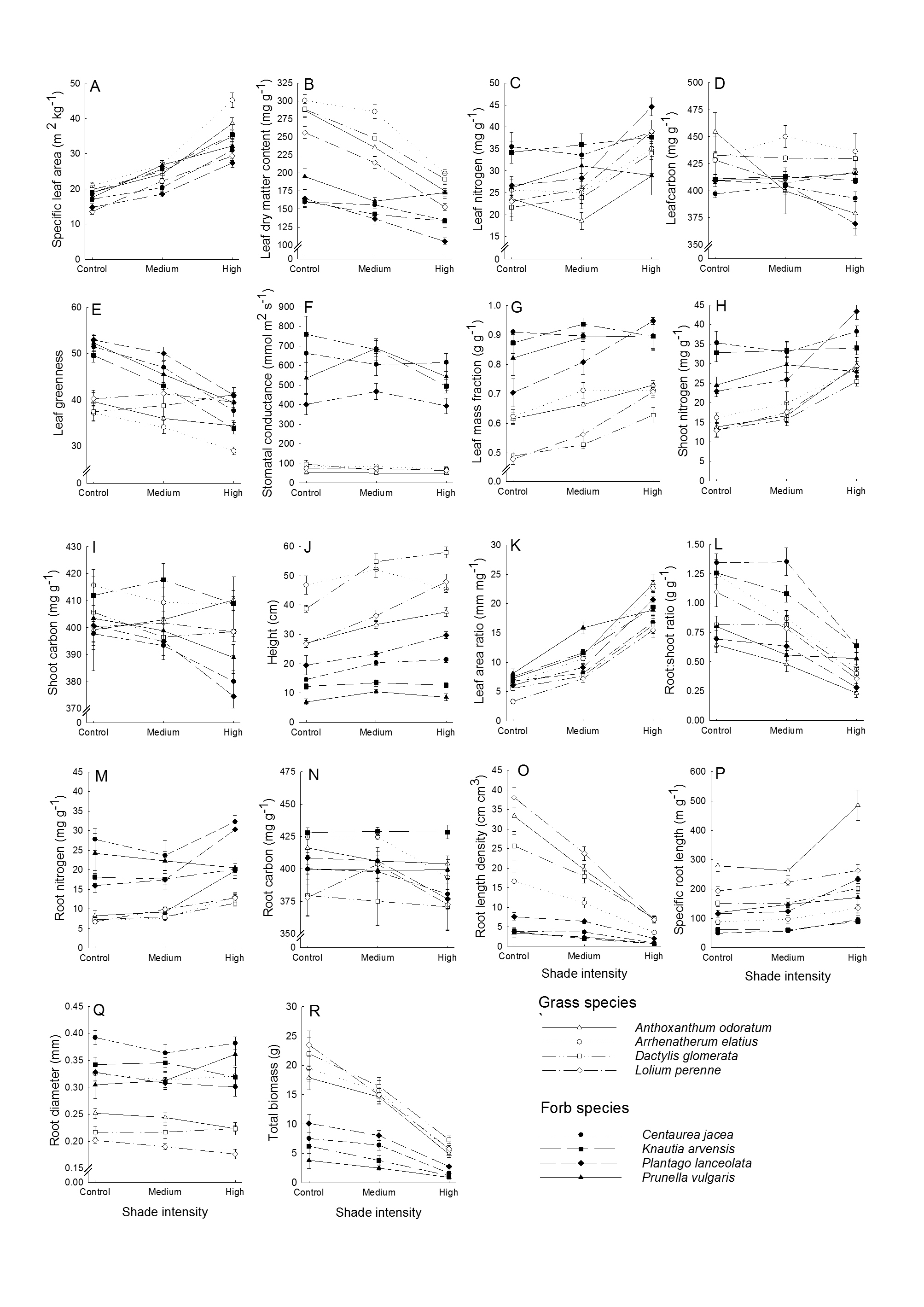
Figure S1. Specific leaf area (A), leaf dry matter content (B), leaf nitrogen concentration (C), leaf carbon concentration (D), leaf greenness (E), stomatal conductance (F), leaf mass fraction (G), shoot nitrogen concentration (H), shoot carbon concentration (I), plant height (J), leaf area ratio (K), root to shoot ratio (L), root nitrogen concentration (M), root carbon concentration (N), root length density (O), specific root length (P), root diameter (Q), and total biomass (R) of eight studied grassland species in response to three different levels of fertilization (control = no fertilizer addition, medium, high). Shown are species mean values (± 1SE) across different levels of shading.


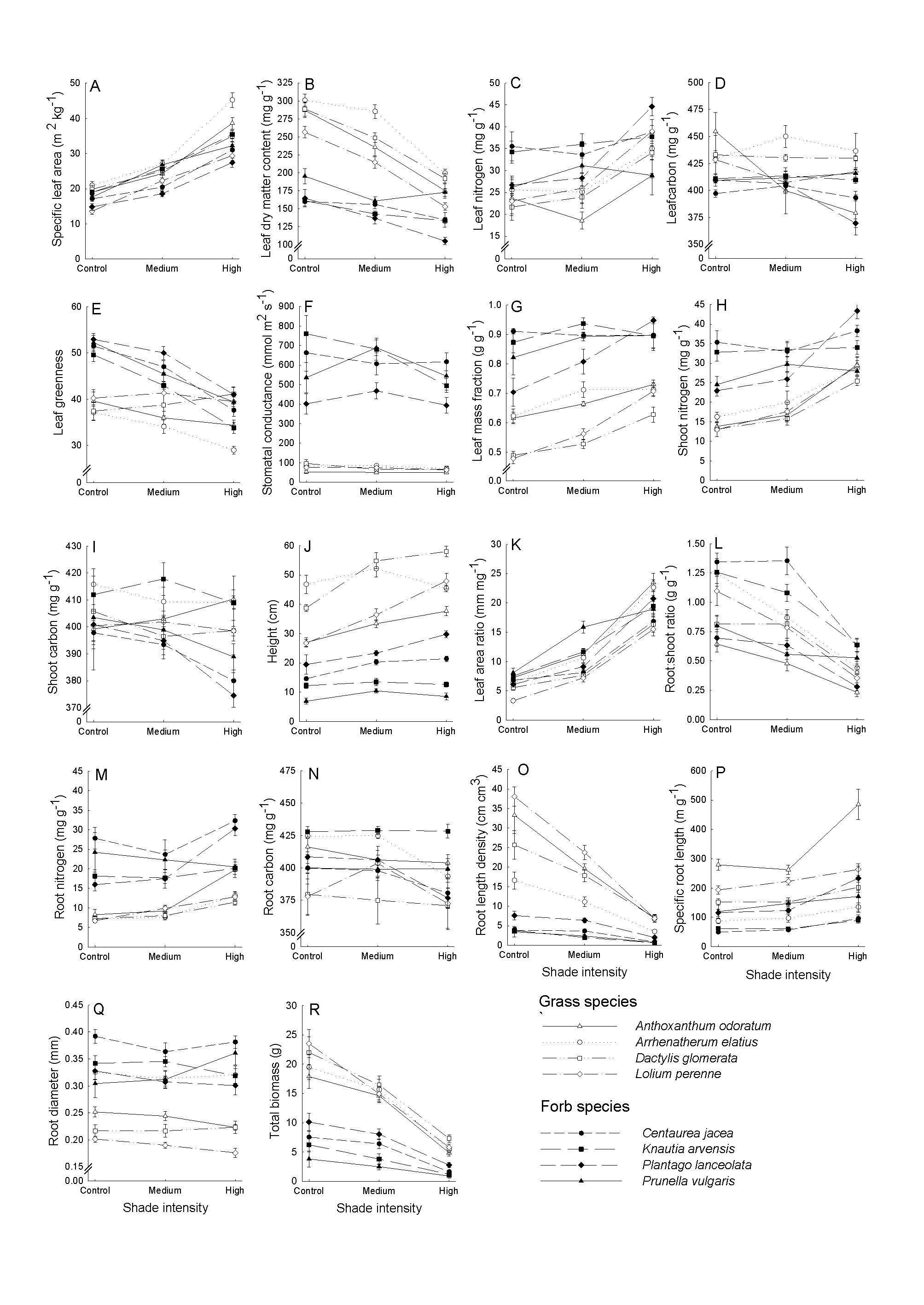


Figure S2. Specific leaf area (A), leaf dry matter content (B), leaf nitrogen concentration (C), leaf carbon concentration (D), leaf greenness (E), stomatal conductance (F), leaf mass fraction (G), shoot nitrogen concentration (H), shoot carbon concentration (I), plant height (J), leaf area ratio (K), root:shoot ratio (L), root nitrogen concentration (M), root carbon concentration (N), root length density (O), specific root length (P), root diameter (Q), and total biomass (R) of eight studied grassland species in response to different levels of shade (control = full light, medium, high). Shown are species mean values (± 1SE) across different levels of fertilization.


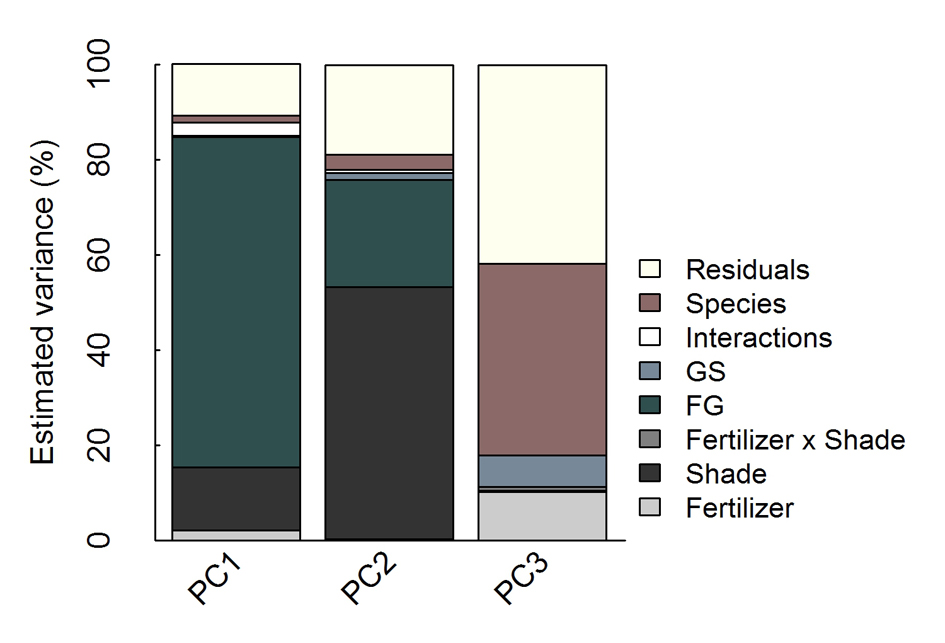


Figure S3. Estimated variance decomposition based on principal components analysis shown in Figure 4. Note that variance components for the interactive effects of FG, GS x resources were combined in the graph as 'interactions').
